# Building Institutional Legitimacy: Elites, Markets, and Inequality Through the Voice–Choice–Transparency–Trust (VCTT) Framework

**DOI:** 10.12688/f1000research.174907.2

**Published:** 2026-05-14

**Authors:** Peter Malliaros, William Alejandro Pacheco-Jaramillo

**Affiliations:** 1 Research Department, UrCommunity Ltda., Melbourne, VIC, 3029, Australia; 2 Economics, University of Canberra Faculty of Business Government & Law, Canberra, Australian Capital Territory, 2617, Australia

**Keywords:** C33 (Panel Data Models), D72 (Political Processes), D73 (Bureaucracy; Corruption), E02 (Institutions and the Macroeconomy), O43 (Institutions and Growth), P16 (Political Economy of Capitalism; Corporate Governance), and H11 (Structure, Scope, and Performance of Government).

## Abstract

**Background:**

Institutional legitimacy—the belief that authorities are just and deserving of compliance—depends on the interplay of participation, pluralism, transparency, and trust. This study applies the Voice–Choice–Transparency–Trust (VCTT) framework to construct a composite institutional quality index (INST) and to examine how elite behaviour, market structure, inequality, and development jointly shape legitimacy across countries.

**Methods:**

Using an unbalanced panel of 22 countries from 2020 to 2024 (T = 5), the study combines hierarchical multiple imputation with panel econometrics. Institutional quality (INST) was derived from equally weighted, standardised VCTT pillars. Core regressors include the Elite Quality Index (EQx), market power (mark-up), the Gini coefficient, and GDP per capita. Two complementary estimators were applied: a pooled panel specification and a Mundlak correlated random-effects model with within–between decomposition, both corrected for heteroskedasticity and cross-sectional dependence using Driscoll–Kraay robust standard errors.

**Results:**

Higher Elite index values are positively associated with institutional quality, whereas greater market concentration is negatively associated with it. The Mundlak model confirms a positive within-country effect of elite quality and a negative within-country effect of rising inequality, while between-country averages suggest that nations with productive elites and moderate inequality sustain stronger institutions.

**Conclusions:**

Across the sample, institutional legitimacy strengthens where elites are value-creating and markets remain competitive. Concentrated market power erodes institutional quality, and inequality’s influence is context-dependent—detrimental when it widens domestically but more neutral when moderate and embedded in stable development trajectories. Because the panel covers only five annual waves, the findings are interpreted as robust associations rather than definitive long-run causal estimates.

## 1. Introduction

Institutional legitimacy—the belief that authorities and institutions are appropriate, just, and worthy of compliance—has long preoccupied political economy and comparative politics. Foundational accounts link legitimacy to norms, legality, performance, and communicative rationality, emphasising that compliance is sustained when institutions are perceived as fair, effective, and inclusive. ^16, 23, 43, 51^ Contemporary comparative data make this insight testable at scale. Measures such as V-Dem, Worldwide Governance Indicators (WGI), and the Corruption Perceptions Index (CPI) have enabled a new generation of empirical studies connecting institutional trust and legitimacy to democratic quality, the rule of law, and state capacity. ^13, 29, 50^

This paper builds a formal institutional index (INST) grounded in the VCTT framework—Voice, Choice (Pluralism), Transparency, and Trust—to model legitimacy as a measurable, multidimensional outcome. We standardise each pillar and combine them with equal weights to capture an unbalanced view of institutional quality while attenuating redundancy across highly correlated inputs. Substantively, INST operationalises a view of legitimacy that is process-based and outcome-observed: societies that protect voice and pluralism and maintain transparency tend to generate higher and more resilient trust. ^57^ Empirically, however, the VCTT pillars are known to move together across countries; in preliminary analyses, we documented high pairwise correlations among Voice, Transparency, and Trust, with Choice showing more limited within-country variation over time. To avoid unstable estimates when using the four pillars jointly as regressors, we treat their standardised aggregate as the dependent variable, thereby shifting the question from “Do V, C, and T predict T?” to the more encompassing “What explains the composite VCTT institutional outcome (INST)?”

We hypothesise that variation in INST reflects deeper structural and policy conditions: the quality of elites, the distribution of income, the degree of market power, the level of development, macroeconomic slack, tertiary education, and R&D intensity. First, the Elite Quality Index (EQx) captures the balance between value-creating and rent-extracting behaviour among elites and the institutional equilibria they sustain. ^12^ Second, inequality—as measured by the Gini coefficient—is linked to lower social and institutional trust and perceptions of unfairness. ^9, 39, 44^ Third, market power—proxied by average mark-ups—relates to economic concentration, potential regulatory capture, and diminished pluralism in both markets and politics. ^14, 15, 49, 55^ Fourth, GDP per capita associates with the resources and demands that support democratic accountability and administrative capability. ^11, 28, 33^ Fifth, unemployment represents macroeconomic distress that plausibly depresses trust in institutions, especially during crises. ^6, 38^ Sixth, tertiary education enrolment or attainment fosters political awareness, engagement, and support for democratic values, correlating with greater institutional support in well-governed contexts. ^10, 21, 25^ Seventh, R&D expenditure as a share of GDP captures innovation capacity, indicating forward-looking economies facilitated by effective governance and associated with enhanced legitimacy. ^3, 5, 19^

This study makes three contributions. First, it proposes a transparent VCTT-based index—INST—constructed from standardised Voice, Choice, Transparency, and Trust, designed for replication and extension. Second, it tests whether EQx, Gini, mark-ups, GDP, unemployment, tertiary education, and R&D explain the cross-national and temporal distribution of INST across an unbalanced panel of 22 countries observed annually from 2020 to 2024 (N = 22, T = 5; up to 110 country–year observations). The five-year horizon is a substantive limitation, not a source of long-run causal certainty; it reflects the common overlap of the VCTT inputs, EQx, mark-up, inequality and macroeconomic controls. For this reason, the empirical strategy emphasises short-panel diagnostics, Mundlak within–between decomposition, Driscoll–Kraay standard errors, imputation checks, PCA sensitivity, lags, subsamples, and leave-one-out checks rather than strong dynamic claims. Third, it connects empirical results to an applications perspective—legitimacy-centred economics—that positions VCTT as a governance backbone for market design and policy instrumentation. Results indicate that value-creating elites are associated with stronger institutional quality, persistent mark-ups with weaker institutions, and inequality with different within- and between-country patterns.

A distinctive contribution of this study is to translate the broad narrative of “inclusive vs. extractive” institutions into an operational and replicable measurement strategy for institutional legitimacy. Rather than stopping at typologies à la Acemoglu and Robinson (2012), ^2^ we construct a composite INST index that embeds Voice, Choice, Transparency, and Trust (VCTT) and make this composite the outcome of interest, explicitly addressing the co-movement among these pillars that complicates inference when they are used as parallel regressors. ^35, 43, 51^ The revised framing also places VCTT beside recent work on state capacity and institutional persistence, including multidimensional state-capacity measurement, dynamic accounts of institutional persistence and change, and panel evidence on the evolution of institutions and development outcomes. ^63– 67^ We then open the black box of legitimacy by integrating three structural drivers that macro-historical accounts often treat qualitatively—elite quality, market power (mark-ups), and income inequality—and by asking whether economic concentration is associated with weaker institutional quality. Substantively, the framework suggests that legitimacy rises where value-creating elites, competitive market design, and equitable outcomes reinforce the VCTT backbone, while its empirical claims remain bounded by the short 2020–2024 panel.

## 2. Theoretical framework

Institutional legitimacy in the VCTT (Voice–Choice–Transparency–Trust) sense hinges on value-creating elites who expand participation (Voice), preserve real options (Choice), institutionalise disclosure and auditability (Transparency), and thereby sustain normative acceptance (Trust). In this sense, institutional legitimacy reflects not only formal rules and performance outcomes but also shared beliefs and perceptions regarding the rightful exercise of authority. ^52^ Procedural-justice research shows that citizens judge authorities as legitimate when decision rules are fair and impartial—conditions that rent-seeking elites routinely undermine (closing voice channels, narrowing choice, and obscuring processes). By contrast, value-creating elites’ lower arbitrariness and aligning incentives with broad public goods, reinforcing impartial administration and social trust. ^43, 51^ At the macro level, the comparative political-economy literature links inclusive, non-extractive elites to institutional arrangements that foster innovation and shared prosperity, whereas extractive elites entrench privileges that erode both performance and consent. ^2, 42, 59^ These dynamics map directly onto VCTT: where elites invest in openness and contestation, legitimacy becomes self-reinforcing; where they extract, every pillar weakens.

Institutional legitimacy (VCTT) is also theorised to depend on core socioeconomic factors, which motivates including GDP per capita, tertiary education, income inequality, market power (markup), and R&D intensity as key independent variables. Each captures a distinct dimension that can shape public perceptions of institutional fairness and performance. Economic development, proxied by GDP per capita, is classically linked to legitimacy: greater prosperity tends to bolster citizens’ confidence in institutions by fulfilling material expectations and enabling better governance capacity. ^33^ Cross-national evidence indeed shows that wealthier societies report higher trust in government. ^11^ Human capital, measured by tertiary education enrolment or attainment, is included because an educated populace is more politically aware and engaged, which can strengthen legitimacy through demand for accountability and support for democratic values. Higher education levels correlate with greater institutional support in well-governed contexts, ^25^ echoing modernization theory that development (income and schooling) underpins stable, legitimate democracy. ^33^ The Gini coefficient represents income inequality – a critical factor because large disparities can undermine the perceived fairness of the system. Conceptually, when economic outcomes are very unequal, citizens may view institutions as serving only elites, eroding institutional trust. Empirical studies confirm that higher inequality is associated with lower confidence in state institutions. ^39, 43^ We also include a markup indicator (e.g. average price-cost margin) as a proxy for market competitiveness and power concentration. This reflects the idea that market structure and institutional integrity are intertwined: if a few firms can consistently charge high markups, it may signal weak competition policies or regulatory capture, which could breed public scepticism about institutional efficacy. Recent research in political economy suggests rising market power can reduce trust in institutions and drive political discontent. ^14^ Finally, R&D expenditure (as a share of GDP) is incorporated to capture the innovation capacity of the economy. R&D investment indicates a forward-looking, knowledge-based economy often facilitated by effective governance. ^19^ It is conceptually relevant because societies that invest in innovation and technology tend to have more modern, responsive institutions, which can enhance legitimacy. In sum, these five predictors cover prosperity, human development, social equity, market fairness, and future-oriented capacity – all factors grounded in theory and evidence as influences on institutional legitimacy and public trust in governance. By including these, the model acknowledges that legitimacy is not only a political or procedural outcome ^45^ but is also shaped by the economic and social context in which institutions operate. ^8, 33, 43^ This perspective is consistent with approaches that emphasise not only input and output legitimacy, but also the quality of governance processes themselves (“throughput legitimacy”), including transparency, accountability, and procedural fairness. ^46^

Classic theories understand legitimacy as a foundation of political order: individuals comply with institutions when they believe the rules are rightful and fairly administered. ^16, 51, 53^ Legitimacy may derive from procedures (voice, representation), norms (fairness, impartiality), or performance (effective service delivery), and it is reinforced by transparent processes that enable accountability. ^35, 43^ In empirical literature, institutional trust features as both an outcome and a driver: it is shaped by experiences with the state and, reciprocally, conditions the effectiveness of policy and the resilience of democratic norms. ^13, 29, 44^

Recent measurement advances allow multi-dimensional operationalisations of democratic quality and governance, including voice and accountability, political competition, transparency and control of corruption, and public trust. These measures reveal strong co-movement across high-income democracies, consistent with the view that open, accountable institutions tend to co-evolve. ^28, 35^ Yet the same co-movement complicates econometric identification: when Voice, Choice, Transparency, and Trust are placed together as regressors, multicollinearity inflates uncertainty. This motivates our modelling choice: we treat a standardised aggregate of the four pillars—INST—as the dependent variable, enabling a sharper test of what drives institutional legitimacy across time and space.

A growing body of work emphasises that who governs—and how elites structure the economy and the state—has first-order implications for institutional outcomes. ^2, 57^ The Elite Quality Index (EQx) synthesises indicators of value creation vs. rent extraction, competition vs. cronyism, and human capital vs. capture, yielding an annual cross-country metric. ^12^ Conceptually, higher EQx signals stronger constraints on predation and greater elite incentives to support impartial administration, open competition, and a credible rule of law—conditions conducive to transparency, pluralism, and trust. ^62^

Scholars in development economics and institutional political science alike have analysed the distinction between extractive and value-creating forms of power, emphasising its impact on long-run economic outcomes. ^2, 36^ Extractive power denotes institutional configurations that enable elites to appropriate resources from the broader population, concentrating wealth and decision-making in ways that stifle innovation and broad-based growth. ^2^ This dynamic reflects North’s (1990) insight that institutions effectively set the “rules of the game”: when those rules are structured for elite rent extraction rather than inclusive opportunity, productive incentives and investment are undermined. By contrast, value-creating power is embodied in inclusive institutions that channel authority toward providing public goods, securing property rights, and expanding opportunities for society at large. ^18^ Such inclusive arrangements encourage entrepreneurship and human capital development across society, establishing a positive-sum dynamic of innovation and shared prosperity. ^2^

Beyond economic performance, the extractive–inclusive dichotomy has critical implications for political legitimacy and social trust. Extractive institutions often undermine political legitimacy: by privileging narrow elites and tolerating high levels of coercion or corruption, they erode public trust in authorities. ^31, 43^ When power is exercised in a predatory manner, citizens become less willing to comply with laws or finance public goods, weakening the social contract. ^31^ In contrast, value-creating power bolsters legitimacy by demonstrating that institutions serve the common good, thereby strengthening institutional trust and social cooperation. ^18, 32^ Governments that deliver broad-based benefits and uphold impartial rules foster a virtuous cycle in which trust and economic inclusion reinforce inclusive governance and equitable development. ^17, 27, 43^

Income inequality significantly undermines institutional trust by eroding perceptions of fairness, shared citizenship, and the normative foundations of legitimate governance. ^44^ Cross-national research consistently reports robust negative associations between income inequality, as measured by the Gini coefficient, and both trust in government and generalised social trust, with panel designs strengthening causal claims by leveraging temporal variation. ^9^ This erosion is not merely perceptual but deeply structural, as inequality maps onto political representation asymmetries and policy capture, ^61^ where elites disproportionately influence outcomes, weakening the perceived responsiveness critical to the voice component of the VCTT framework. ^20^ Moreover, inequality is often perpetuated by extractive institutions—structures that concentrate power and wealth among elites, as described by Acemoglu and Robinson (2012), ^2^ who argue that such systems prioritise elite interests over broad societal welfare, inherently destabilising legitimacy. For these elites, maintaining or widening income gaps can be a strategic “good business,” as it reinforces their control over resources and political processes, entrenching power imbalances that further alienate citizens. ^40^ This dynamic is particularly pronounced in contexts where economic elites leverage inequality to secure favourable policies, such as tax exemptions or deregulation, which exacerbate distrust by signalling that institutions serve the few rather than the many. ^24^ Consequently, inequality not only undermines the procedural fairness and inclusivity central to legitimacy but also fuels a vicious cycle where extractive power structures deepen public disillusionment, reducing compliance and eroding the diffuse support necessary for stable democratic governance. ^16^

The rise of mark-ups and “superstar” firms has shifted focus to market concentration and its implications for economic and political power, highlighting how a handful of dominant players can reshape entire industries. ^7, 15^ Classic political economy suggests that concentrated market power fosters regulatory capture and rent-seeking, consolidating influence among elites who manipulate rules to their advantage. ^49^ When a few dominant firms control markets, they can skew economic opportunities, narrow pluralism, and enable disproportionate lobbying that undermines regulatory impartiality and distorts fair competition. ^55^ This dynamic erodes transparency, as powerful actors obscure rent-seeking practices through opaque influence, weakening the perceived fairness of institutions critical to legitimacy. ^2^ Moreover, entrenched market power often benefits elites by perpetuating wealth disparities and reinforcing extractive structures that prioritise their interests over broader societal welfare, creating barriers to entry that stifle innovation and mobility. ^40^ In this way, high mark-ups not only signal economic inefficiency but also perpetuate cycles of inequality, where elites view concentration as a strategic tool to maintain control, further alienating citizens and eroding trust in impartial governance. Consequently, rising mark-ups act as a macro-level indicator of market structure distortions, directly linked to diminished institutional legitimacy by compromising voice, pluralism, and transparent governance.

The starkest examples of extractive power dynamics are evident in developing countries, where commodity-driven economies often exacerbate the concentration of wealth and political influence, perpetuating cycles of underdevelopment. ^2^ In resource-rich nations like those in Latin America or sub-Saharan Africa, the “commodities business” generates market concentration as elites capture rents from exports such as oil, minerals, or agriculture, leading to the Dutch disease phenomenon—where resource booms appreciate currencies, hollow out non-resource sectors, and stifle diversification. ^60^ This not only distorts economic pluralism by favouring extractive industries but also intensifies rent-seeking, where powerful actors manipulate institutions to secure privileges, further eroding transparency and institutional legitimacy. ^59^ However, addressing this is not merely a matter of creating incentives for power holders to shift from value extraction to value generation; strong institutions are essential to curb elite capture and foster inclusive growth. Yet, this raises a potential vicious circle: do robust institutions precede and constrain extractive power, or must power first establish mechanisms for institutional strengthening? It seems more plausible that entrenched power, when aligned with reformist incentives or external pressures, can initiate the creation of accountability mechanisms—such as independent judiciaries or anti-corruption bodies—to gradually build institutionality, breaking the cycle through deliberate elite-led transitions rather than waiting for spontaneous institutional emergence. ^36^

Economic development, often proxied by log GDP per capita, not only correlates with stronger institutional quality but also engages in a bidirectional relationship where improved institutions can drive further growth, creating a virtuous cycle of progress. ^28, 33, 35^ As economies advance, rising GDP per capita expands the resource base for public investments, heightens societal demands for accountable governance, and fosters expectations for high-quality services, all of which bolster stable democracy and institutional trust by enabling more inclusive and effective administration. Conversely, superior institutions—characterised by secure property rights, rule of law, and reduced corruption—facilitate economic growth by encouraging investment, innovation, and efficient resource allocation, as evidenced in empirical studies showing that institutional reforms precede sustained GDP increases (Acemoglu et al., 2001). This interplay is particularly evident in transitions from low- to middle-income statuses, where institutional strengthening mitigates risks like rent-seeking and policy volatility, unlocking productivity gains. ^42^ However, macroeconomic slack, such as high unemployment, introduces distress that depresses trust, amplifying perceptions of institutional failure during downturns and crises by straining public resources and exacerbating social divides. ^6, 38^ Taken together, these factors articulate a structural account: elites, distributional fairness, market openness/competition, state capacity, and macro performance jointly shape the institutional equilibrium reflected in INST, with the bidirectional link between growth and institutions underscoring the need for targeted reforms to break potential vicious cycles in underperforming economies.

Tertiary education expansion is widely linked to stronger governance and higher institutional legitimacy. An educated citizenry demands transparency, accountability, and quality public services, reinforcing compliance based on perceived fairness rather than coercion. ^33^ Tertiary education disproportionately supplies the civil service, judiciary, media, and regulatory professions with advanced analytical skills, professional norms, and policy literacy that directly underpin state capacity and the rule of law. Relative to earlier schooling, universities generate research, expert networks, and civic leadership that diffuse accountability standards across public and private organisations, making institutional improvements more likely and durable. Cross-country evidence associates higher educational attainment with democratic development and improved institutional performance. ^10, 21^ Human capital and institutions co-evolve. Better schooling raises civic and economic capabilities that, in turn, sustain impartial, rules-based governance. ^26^ As tertiary attainment diffuses, organisational capacity and political voice extend beyond traditional elites, weakening rent-seeking coalitions and shifting power toward more inclusive, value-creating equilibria. ^2, 37, 60^ In this sense, university education not only augments skills but also broadens the social base that monitors and constrains authority, bolstering legitimacy through participation, pluralism, and credible oversight. ^26, 33^

National investment in research and development (R&D) similarly shapes legitimacy and the distribution of power by catalysing innovation and structural change. Schumpeterian growth theory posits that R&D drives “creative destruction,” reallocating resources from incumbents to more productive firms and sectors. ^3, 47^ When institutions are inclusive, innovation intensifies competition, diffuses economic opportunities, and undercuts extractive rents—mechanisms that reinforce transparency, choice, and trust. ^4, 42^ Conversely, elites may resist innovation when it threatens entrenched privileges—the “fear of creative destruction”—with predictable consequences for governance quality. ^2^ Empirical syntheses show that sustained R&D effort, combined with open and contestable markets, is associated with faster productivity growth and broader welfare gains, conditions that elevate institutional performance and perceived legitimacy. ^5, 41^ In short, higher R&D spending can shift economies—and power structures—away from rent extraction toward value creation, provided the institutional environment protects entry, enforces fair rules, and channels technological change into inclusive outcomes. ^4, 42^

## 3. Methods

This study combines hierarchical multiple imputation with the Mundlak correlated random-effects framework, allowing the use of all available countries despite missing data. The approach preserves both within- and between-country variation while reducing bias from unobserved country heterogeneity. Driscoll–Kraay robust standard errors are used to guard against heteroskedasticity and cross-sectional dependence. Because the panel is short (22 countries observed over 2020–2024), these techniques strengthen inference for structured associations but do not by themselves establish long-run causality.

The methodology involved a comprehensive econometric approach to explore the relationship between institutional quality (INST) and independent variables such as elite behaviour, market concentration, inequality, and socio-economic factors. The dataset includes an unbalanced panel of 22 countries from 2020 to 2024, sourced from publicly available repositories including the World Bank, V-Dem, WGI, SWIID, the Elite Quality Index, OECD/UNESCO/ILOSTAT series, and De Loecker–Eeckhout–Unger mark-up data. Institutional quality was measured using the VCTT framework (Voice, Choice, Transparency, and Trust), which captures participation, pluralism, transparency, and confidence in institutions.

The dependent variable is a composite index of institutional quality (INST). Key explanatory variables include: (1) an indicator of elite power (Elite), measuring the concentration of political-economic influence; (2) an average profit margin indicator (Markup), an inverse proxy for market competition (higher markups imply less competition and potential market power); (3) the logarithm of real GDP per capita, as a measure of economic development; and (4) the Gini coefficient, as an indicator of income inequality. The selection of these 22 countries, beyond data availability, is crucial for examining the impact of institutional quality on economic outcomes, as it enables us to distinguish between countries with strong institutional frameworks and those with weaker ones ( Table 1). Developed countries, typically characterised by higher institutional quality as reflected in indicators like governance, transparency, and trust, form one group. These nations, such as Germany, the United States, and Canada, are often seen as having resilient and efficient institutions that support sustainable economic growth and social stability. On the other hand, the emerging and developing countries in the sample—such as Brazil, Mexico, and Turkey—tend to have more vulnerable institutional setups, which can be influenced by factors like political instability, inequality, and less robust public trust in governance. By categorising the countries into these two distinct groups, the study seeks to explore how the presence or absence of strong institutions impacts economic performance, particularly in terms of legitimacy, market efficiency, and societal equity. This classification serves as a foundation for understanding the dynamics that shape institutional development across different contexts.

**Table 1.** Descriptive statistics by development level.

Variable	Developed countries		Emerging and developing countries	
	Mean	SD	Mean	SD
**Institutional Quality (INST)**	0.66	0.22	−0.91	0.71
**Elite Index**	62.21	2.23	51.26	7.43
**GDP per capita (USD)**	61,260	13,271	27,438	11,059
**Gini Coefficient**	31.59	4.03	38.70	8.69
**Market Power (Markup)**	1.57	0.13	1.46	0.23
**R&D Expenditure (% GDP)**	2.80	0.89	1.26	0.74
**Schooling (Mean Years)**	83.42	13.23	64.89	27.71
**Unemployment Rate (%)**	5.13	1.87	10.63	9.99
**Year (mean sample)**	2022.00	1.42	2022.00	1.43

Source: Authors’ based on R estimations.

Institutional Quality (INST) is a standardised z-score composite derived from the Voice–Choice–Transparency–Trust (VCTT) pillars, where positive values indicate above-average institutional legitimacy and negative values denote weaker institutional performance. The Elite Index (EQx) is measured on a 0–100 scale, where higher values reflect value-creating elites and lower scores indicate extractive or rent-seeking elites. ^12^ The Gini coefficient is expressed on a 0–100 scale and represents income inequality; higher values denote greater concentration of income. ^48^ Market Power (Markup) is defined as the ratio of price to marginal cost (P/MC), with values above 1 indicating higher concentration and reduced competition. ^15^ Schooling (Scho) refers to gross tertiary-education enrolment (% of the population aged 18–24) or, where unavailable, mean years of schooling among adults (UNESCO/WB).

The VCTT framework is grounded in the idea that institutional legitimacy arises from the interplay of four key pillars: Voice (effective participation), Choice (pluralism and competitive access), Transparency (observable and accountable processes), and Trust (confidence in the system). These pillars are empirically linked, with the strength of one often enhancing the others. For example, voice and pluralism cannot be maintained without transparency, and transparency itself improves when voice curtails arbitrary decision-making. Ultimately, trust emerges because of these factors working together. ^35, 43, 51^ This conceptual synergy often leads to multicollinearity when these factors are entered simultaneously into the regression model, requiring careful treatment of variables to ensure robust analysis.

Operationally, INST was constructed in three steps. First, each VCTT pillar was harmonised to the country–year panel and oriented so that higher values indicate stronger legitimacy. Second, each pillar was standardised as a z-score over the analytical sample. Third, the four standardised pillars were averaged with equal weights and re-standardised to mean zero and unit variance. Equal weighting is used because the VCTT model treats the pillars as mutually reinforcing dimensions rather than a ranked hierarchy; as a robustness check, a PCA-based composite was also examined and did not alter the substantive direction of the results.

Missing values were treated using hierarchical multiple imputation rather than listwise deletion. The imputation model included country and year structure, the four VCTT pillars, EQx, Gini, mark-up, GDP per capita, unemployment, schooling and R&D. Multiple completed panels were generated, estimates were pooled using standard multiple-imputation rules, and observed-versus-imputed distributions were inspected before estimation to ensure that central tendency, dispersion and plausible country-year variation were preserved.

To supplement the regression analysis, an ARIMA exercise was used only as a sensitivity and projection tool for the mark-up series. Historical mark-up observations were modelled and projected through 2024 to assess whether the direction of market concentration remained consistent with the panel estimates. Model orders were selected using information criteria and residual diagnostics. These projections are not treated as additional observed panel data and are not used to make causal claims; they provide a robustness-oriented view of how market concentration may evolve under recent trends.

Descriptive correlations among the main variables are reported in Table 2. The matrix captures the direction and strength of associations between institutional, economic, and social indicators, providing a preliminary view of potential linkages before multivariate estimation.

**Table 2.** Correlation matrix of key variables.

Variable	Year	GDP	R&D	Unemployment	Schooling	Gini	INST	Elite	Markup
**Year**	1.00	0.07	–0.08	–0.07	0.04	0.03	0.00	0.05	0.07
**GDP per capita**	0.07	1.00	0.51	–0.50	0.51	–0.59	0.78	0.76	0.24
**R&D expenditure (% GDP)**	–0.08	0.51	1.00	–0.38	0.45	–0.30	0.39	0.65	–0.34
**Unemployment rate (%)**	–0.07	–0.50	–0.38	1.00	–0.48	0.49	–0.14	–0.66	–0.67
**Schooling (education index)**	0.04	0.51	0.45	–0.48	1.00	–0.21	0.42	0.39	–0.53
**Gini coefficient**	0.03	–0.59	–0.30	0.49	–0.21	1.00	–0.45	–0.74	–0.13
**Institutional Quality (INST)**	0.00	0.78	0.39	–0.14	0.42	–0.45	1.00	0.51	0.17
**Elite Index**	0.05	0.76	0.65	–0.66	0.39	–0.74	0.51	1.00	0.47
**Market Power (Markup)**	0.07	0.24	–0.34	–0.67	–0.53	–0.13	0.17	0.47	1.00

Source: Authors’ calculations based on R estimations.

The correlation structure indicates several theoretically consistent patterns. Institutional quality (INST) is strongly and positively correlated with GDP (r = 0.78) and Elite (r = 0.51), suggesting that higher economic development and value-creating elites are associated with stronger institutions. Income inequality (Gini) shows a negative relationship with INST (r = –0.45) and Elite (r = –0.74), confirming that inequality erodes both institutional legitimacy and elite quality. As expected, unemployment is inversely correlated with GDP (r = –0.50) and education (r = –0.48), while R&D and schooling exhibit positive associations with economic performance and elite quality. The relatively low correlations between Markup and the other institutional variables (|r| < 0.5) suggest that market power operates as an independent dimension of institutional legitimacy rather than being collinear with development or inequality.

**Figure 1.**
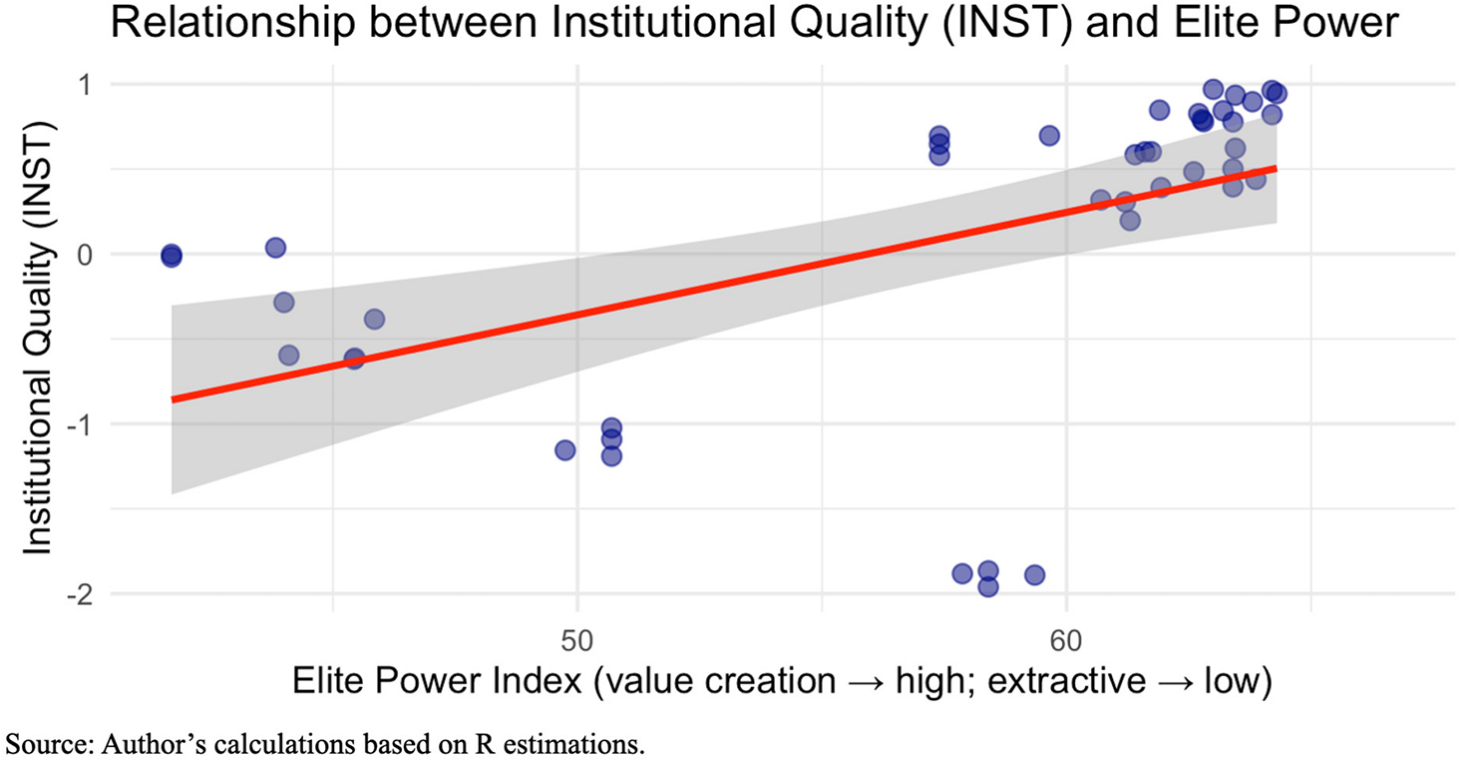
Relationship between elite power and institutional quality. Source: Author’s calculations based on R estimations.

Figure 1 shows the scatter plot displaying the association between the Elite Power Index and Institutional Quality (INST) across all available countries. Higher values of the Elite index indicate more productive, value-creating elites, whereas lower values reflect more extractive elite structures. The positive slope of the fitted regression line (in red) suggests that, in this sample, countries with more value-oriented elites tend to exhibit stronger institutional quality.

The variable Elite captures the influence of economic/political elites exhibiting extractive behaviour in each country-year, whereas Markup approximates average market power (e.g., price–cost margins) as an indicator of competition or market concentration. The controls help isolate the effects of Elite and Markup on institutional quality, acknowledging, for example, that higher inequality may tilt outcomes toward lower-quality institutions ^1^ and that human capital and macroeconomic conditions shape institutional performance.

The general econometric specification is:

$$\mathrm{INS}T_{i,t}=\alpha +\beta_{1}\;\text{Elit}e_{i,t}+\beta_{2}\;\text{Marku}p_{i,t}+\beta_{3}\;{\text{Gini}}_{i,t}+\gamma^{\prime }X_{i,t}+\mu_{i}+\lambda_{t}+\varepsilon_{i,t},$$

where i indexes countries ( i=1,…,22 ) and t indexes years ( t=1,…,5 ). The vector $X_{i,t}$ collects the controls (Gini, unemployment, schooling, etc.); $\mu_{i}$ captures unobserved, country-specific factors (e.g., historical or cultural influences on institutions); and $\lambda_{t}$ are time fixed effects (to net out year-specific common shocks, when relevant). The idiosyncratic error term is $\varepsilon_{i,t}$ . Because $\mu_{i}$ may correlate with observables, we estimate both random-effects (RE) and fixed-effects (FE) models. The RE estimator assumes $\mu_{i}$ is independent of the regressors, exploiting both between- and within-country variation, while FE relaxes this assumption and uses only within-country variation over time.

The sample is potentially unbalanced (some countries lack data for certain years); therefore, methods that utilise all available information without discarding cases due to missing values are employed. ^22, 30, 54^ Some collinearities between Gini and GDP per capita were detected (wealthier countries typically exhibit lower inequality); therefore, inequality coefficients are interpreted cautiously when controlling for development level.

For econometric estimation, a random effects (GLS) model with Amemiya’s (1971) transformation was employed, which subtracts from each observation a fraction of the country mean, attenuating the influence of unobserved fixed factors. This approach leverages variation both between countries and over time. However, if the unobserved effects of each country correlate with the regressors, RE estimates would be inconsistent (Hausman, 1978). It is plausible that historical or institutional characteristics of each nation are associated with, for example, elite power or inequality.

To relax this assumption without losing between-unit variation, the Mundlak (1978) specification is adopted, adding country means of time-varying explanatory variables as regressors. By including these averages ${\overset{-}{X}}_{i}$ alongside deviations ( $X_{\text{it}}-{\overset{-}{X}}_{i}$ ), the model controls for unobserved country-level heterogeneity potentially correlated with the regressors. This “correlated random effects” approach produces estimates equivalent to a fixed effects model for the included variables but preserves countries with incomplete data and improves efficiency by exploiting between-unit information.

Additionally, standard errors robust to heteroskedasticity and cross-sectional dependence are calculated using the Driscoll and Kraay (1998) matrix. This correction supports more reliable inference when common shocks affect countries simultaneously. Given the short time dimension of the panel, however, Driscoll–Kraay estimates are interpreted cautiously and are reported alongside sensitivity checks. In sum, the random-effects model with Mundlak adjustments and robust errors helps separate within-country from between-country associations while reducing bias from unobserved country-level heterogeneity.

Endogeneity is addressed as a central limitation rather than treated as fully solved. Elite structure, market power and institutional legitimacy may influence each other: stronger institutions can discipline elites and markets, while value-creating elites and competitive markets can strengthen institutions. The Mundlak decomposition reduces bias from time-invariant country heterogeneity, lagged and leave-one-out checks test sensitivity, and the interpretation therefore emphasises conditional associations and plausible mechanisms rather than one-directional causality.

## 4. Results

Table 3 reports the results from the pooled OLS estimation with Driscoll–Kraay robust standard errors. The model shows that the Elite coefficient is positive and highly significant ( *p* < 0.01), indicating that countries with more productive and value-creating elites tend to achieve higher institutional quality. In contrast, the Markup variable has a large and strongly negative effect ( *p* < 0.01), suggesting that greater market concentration is associated with weaker institutions, likely reflecting reduced competition and possible elite capture. The coefficient for Gini is positive and significant at the 5 per cent level, implying that in certain contexts moderate inequality can coexist with institutional stability—possibly a reflection of structural or developmental factors rather than a causal improvement. Overall, the results emphasise the dual nature of economic power: while productive elites can strengthen institutions, excessive market dominance erodes them.

**Table 3.** Regression results from fixed effects model (Pooled OLS with Driscoll-Kraay robust standard errors).

Variable	Estimate	Signif.	Economic interpretation
Intercept	5.255	***	Baseline institutional quality level when all predictors are at their mean.
Elite	0.143	***	A higher Elite index—representing more productive, value-creating elites—is strongly associated with improved institutional quality.
Markup	–8.351	***	Greater market concentration (higher mark-ups) substantially weakens institutional quality, reflecting potential capture and reduced competition.
Gini	+0.012	**	Moderate inequality appears positively related to institutional quality, possibly capturing effects of development level or institutional adaptation.

Source: Authors’ calculations based on R estimations.

*** p < 0.01,

** p < 0.05,

* p < 0.1.

**Figure 2.**
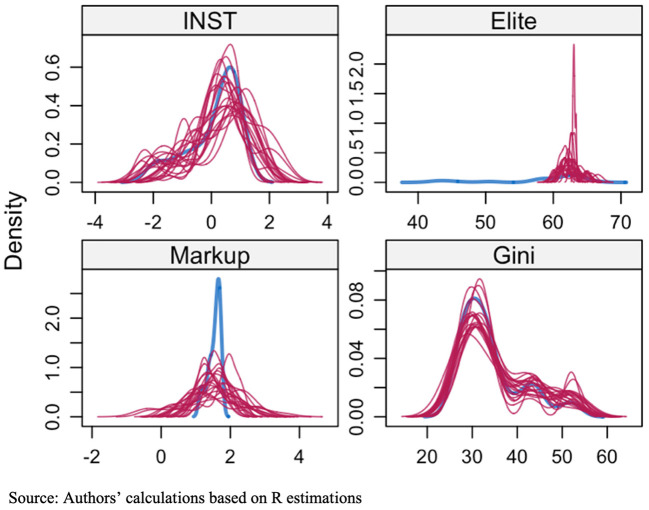
Density distributions of imputed and observed variables (INST, Elite, Markup, and Gini). Source: Authors’ calculations based on R estimations.

Figure 2 displays the kernel density distributions of the main variables after the multiple imputation process, comparing the variability among the imputed datasets (in magenta) with the original observed data (in blue). For INST, the imputed densities almost perfectly overlap with the original distribution, indicating that the imputation preserved the overall shape and dispersion of institutional quality values. In the case of Elite, the imputed series shows slightly greater variability but remains centred around the same range as the observed data, suggesting that missing values were corrected without distorting the underlying distribution. The Markup variable exhibits a similar pattern: the imputed densities maintain the symmetry and kurtosis of the original data, confirming internal consistency. Finally, Gini shows a somewhat wider spread across imputations, reflecting a higher share of missing observations in this variable; however, the overlapping curves indicate that the imputation retained its main trend and multimodal structure. Overall, the Figure 2 demonstrates that the multiple imputation procedure was statistically appropriate and did not introduce visible biases, supporting the reliability of the completed unbalanced panel used for subsequent econometric estimation.

Table 4 presents the results of the Mundlak model estimated using random effects with a within–between decomposition and Driscoll–Kraay robust standard errors. The findings clearly distinguish between short-run (within-country) and long-run (between-country) dynamics. The within-country component for Elite (Elite_w) is positive and statistically significant ( *p* < 0.01), indicating that improvements in the productivity and inclusiveness of elite groups over time within a country enhance institutional quality. In contrast, Markup_w is negative but insignificant, suggesting that short-term variations in market concentration do not have an immediate measurable effect on institutional outcomes. The Gini_w coefficient is negative and significant ( *p* < 0.05), implying that rising inequality within countries undermines institutional cohesion and erodes governance quality.

**Table 4.** Mundlak model results (Random effects with within transformation) Driscoll-Kraay robust standard errors.

Variable	Estimate	Std. Error	t-value	p-value	Significance
Intercept	1.81411	0.86329	2.10	0.073	·
Elite_w	0.07783	0.01916	4.06	0.004	**
Markup_w	-1.86342	2.63316	-0.71	0.502	
Gini_w	-0.02621	0.01084	-2.42	0.046	*
Elite_mean	0.19459	0.01420	13.70	<0.001	***
Markup_mean	-8.67547	0.57550	-15.07	<0.001	***
Gini_mean	0.04255	0.00723	5.88	<0.001	***

Source: Authors’ calculations based on R estimations.

At the between-country level, the results show that Elite_mean is strongly positive and highly significant ( *p* < 0.001), confirming that countries with more value-generating and non-extractive elites tend to sustain stronger, more legitimate institutions. Conversely, Markup_mean is large, negative, and highly significant ( *p* < 0.001), reinforcing the idea that persistent market dominance and concentration are detrimental to institutional development. Interestingly, Gini_mean shows a positive and significant relationship, suggesting that in more developed economies, moderate levels of inequality may coexist with institutional stability—possibly reflecting a functional balance between incentive structures and social cohesion. Overall, the results support a nuanced interpretation: while productive elites contribute to institutional legitimacy both within and across countries, entrenched market power and widening inequality weaken these foundations, highlighting the importance of maintaining competitive markets and inclusive governance.

## 5. Conclusions

The econometric analysis employed a pooled panel specification and a Mundlak correlated random-effects model, both estimated with Driscoll–Kraay robust standard errors to account for heteroskedasticity, serial correlation, and cross-sectional dependence in a short panel. 56 Together, these complementary approaches provide a consistent picture of the institutional mechanisms operating across and within countries during 2020–2024, while the limited time span means that the estimates should be read as short-run panel associations rather than a definitive long-run verdict.

The results from the Fixed Effects model ( Table 3) show that Elite exerts a strong and highly significant *positive* effect on institutional quality ( *p < 0.01*). This implies that higher Elite index values—indicating more productive and value-creating elites rather than extractive ones—correspond with stronger institutions. Countries where elite groups contribute to innovation, competition, and rule enforcement tend to build institutions with greater transparency and accountability. In contrast, the Markup coefficient is large, negative, and highly significant ( *–8.35; p < 0.01*), signalling that concentrated market power substantially undermines institutional quality. Economically, this reflects how monopolistic structures weaken the checks and balances that sustain trust and contestability. The Gini coefficient displays a modest but statistically significant *positive* effect ( *p < 0.05*), suggesting that moderate inequality can coexist with institutional strengthening, potentially capturing a stage of structural maturity in some advanced economies where institutional frameworks stabilise despite unequal outcomes.

The Mundlak model ( Table 4) allows a richer decomposition between *within-country* and *between-country* dynamics. The within-country Elite effect (Elite_w) remains positive and significant ( *p = 0.004*), confirming that institutional performance improves when elites become more productive and inclusive over time. Conversely, Markup_w remains negative though statistically insignificant, implying that short-term variations in market concentration do not immediately alter institutional outcomes. Gini_w, however, turns negative and significant ( *p = 0.046*), showing that rising inequality within a country erodes institutional quality and legitimacy. On the structural dimension, Elite_mean and Gini_mean are both positive and highly significant ( *p < 0.001*), while Markup_mean remains large and negative ( *p < 0.001*). This combination implies that nations historically characterised by value-creating elites and moderate, long-term inequality exhibit stronger institutions, whereas persistent concentration of market power continues to weaken them.

Taken together, these results show that institutional legitimacy is shaped jointly by political and economic structures. Productive elites enhance institutional performance both over time and across countries. Market concentration consistently damages institutional credibility, and inequality plays a nuanced role: harmful when it widens domestically, but neutral or slightly beneficial when it remains moderate and embedded in stable development trajectories. In essence, balanced power relations—economic and political—create the structural conditions under which institutions can remain both efficient and legitimate. ^56^

### 5.1 Public policy implications

First, it is essential to limit state capture by elites through reforms that strengthen institutional checks and balances and accountability: reinforce judicial independence and oversight bodies and establish strict rules on political financing and conflict of interest to reduce undue influence (Stiglitz, 2012). In parallel, citizen voice in decision-making should be expanded to counter oligarchic power: through effective participation mechanisms (public consultations, participatory budgeting), a free press, and an empowered civil society ensuring policies reflect broader interests, not just those of elites.

Second, competitive and open markets should be promoted. More vigorous anti-monopoly policies, elimination of artificial barriers to new entrants, and support for innovation will help reduce concentration and excessive margins. A more competitive market not only improves efficiency but also dilutes the lobbying capacity of dominant actors, aligning with the “choice” pillar of the VCET framework by providing more options to citizens and entrepreneurs.

Third, reinforcing transparency and institutional integrity is a priority. Adoption of open government practices (publication of open data, online tendering, asset declarations of officials), alongside robust access to information laws and whistleblower protection, reduces spaces for corruption and hinders institutional capture (World Bank, 2017).

Finally, addressing inequality gaps is necessary. Progressive redistributive policies and social investment (education, health, social protection) not only promote greater distributive justice but also strengthen social cohesion and trust in institutions, generating virtuous cycles of legitimacy. ^44^

These measures address the identified distortions: they disperse excessive elite power, maintain market discipline, ensure transparency, and foster social inclusion. International organizations concur in recommending such reforms – the World Bank (2017), for example, emphasises aligning incentives of powerful actors with public interest through legal frameworks that hinder capture. Ultimately, building institutional legitimacy requires combining political and economic changes that limit the discretionary power of elites while promoting market competition and social inclusion, thereby reinforcing the pillars of Voice, Choice, Transparency, and Trust in the long term.

## Data Availability

All data used in this study were obtained from publicly available third-party sources and can be accessed by readers and reviewers through the same procedures used by the authors. Institutional quality (INST) was constructed as a standardized composite of the Voice, Choice, Transparency, and Trust pillars using indicators from the Varieties of Democracy (V-Dem) dataset, available at: https://www.v-dem.net/data/ and the Worldwide Governance Indicators (WGI), available at: Home | Worldwide Governance Indicators Elite characteristics were measured using the Elite Quality Index (EQx) published by the University of St. Gallen, available at: https://elitequality.org/ Income inequality was captured using the Standardized World Income Inequality Database (SWIID), available at: https://fsolt.org/swiid/ Market power was proxied by average mark-ups using the publicly available series developed by De Loecker, Eeckhout, and Unger, available at: https://sites.google.com/site/deloeckerjan/data-and-code - (Aggregate markups from Global Market Power: Country-year (xls) and Continent-year (xls)).This is a publicly accessible database and not a DOI-based repository. Additional macroeconomic controls—including GDP per capita, unemployment rates, schooling indicators, and research and development (R&D) expenditure—were obtained from the following publicly accessible databases:
• World Bank World Development Indicators (WDI): https://databank.worldbank.org/source/world-development-indicators
• OECD Statistics: https://stats.oecd.org/
• UNESCO Institute for Statistics: https://uis.unesco.org/
• ILOSTAT (International Labour Organization): https://ilostat.ilo.org/ The analysis relies on an unbalanced panel of 22 countries covering the period 2020–2024, reflecting the common availability of variables across these sources. No primary data were collected, and no proprietary, confidential, or restricted-access datasets were used. All datasets are freely available and can be accessed directly from the official repositories listed above without restrictions or special permissions. To improve reproducibility, the revised manuscript specifies the construction of INST, the standardisation and equal-weighting procedure, the imputation model, and the use of ARIMA projections as a sensitivity exercise. On resubmission, the authors will provide supplementary replication material containing the harmonised country–year panel and code used to construct the final analytical dataset. All materials used in this study are derived entirely from publicly available secondary sources. No primary data instruments (e.g., surveys, interviews, consent forms) were created for this research.
