# Supplementary material for: Building Institutional Legitimacy: Elites, Markets, and Inequality Through the Voice–Choice–Transparency–Trust (VCTT) Framework
Source: F1000Res. 2026 May 14;15:23. Originally published 2026 Jan 7. [Version 2] doi: 10.12688/f1000research.174907.2 (PMC13270191; doi:10.12688/f1000research.174907.2)
Supplement: Peer review history

# Reviewer report: Reviewer response for version 2

**DOI:** 10.5256/f1000research.201165.r487794

**Published:** 2026-06-15

**Authors:** Cong Minh Huynh

**Affiliations:** 1 Eastern International University, Thủ Dầu Một, Thu Dau Mot City, Vietnam

**F1000Research**

**Title: “Building Institutional Legitimacy: Elites, Markets, and Inequality Through the Voice–Choice–Transparency–Trust (VCTT) Framework”**

 The manuscript develops a composite institutional legitimacy index (INST) based on four dimensions - Voice, Choice, Transparency, and Trust (VCTT) - and examines how elite quality, market concentration, income inequality, and development-related factors affect institutional legitimacy across 22 countries from 2020–2024. Using pooled panel regressions and a Mundlak correlated random-effects model, the authors conclude that value-creating elites strengthen institutional legitimacy, market concentration weakens it, and inequality exhibits mixed effects depending on whether variation occurs within or between countries. The study proposes the VCTT framework as a new governance-oriented lens for understanding institutional quality.

 This is an interesting topic. However, I have some comments below.

 1. The manuscript repeatedly presents very strong causal narratives regarding: elite quality → legitimacy, market power → legitimacy, inequality → legitimacy. However, the empirical evidence consists only of a short panel covering 22 countries and 5 years. But the paper makes claims about: institutional evolution, elite capture, legitimacy formation, extractive versus inclusive institutions, long-run governance trajectories. These claims far exceed what the data can support. The manuscript essentially uses a short-term correlation exercise to support a grand theory of institutional development.

 2. A more fundamental problem is that the dependent variable (INST) is conceptually constructed from: Voice, Choice, Transparency, Trust. The authors then argue that productive elites create transparency, pluralism, trust, and participation which are precisely the ingredients used to construct the dependent variable. The manuscript does not convincingly separate institutional legitimacy, democratic quality, governance quality, trust. These concepts become interchangeable throughout the paper. As a result, the theoretical argument risks becoming tautological: Good institutions are measured by good institutions.

 3. VCTT framework is normative rather than explanatory. The framework is presented as a theory, but it functions more as a normative governance model. The manuscript assumes: voice is good, transparency is good, trust is good, pluralism is good. These assumptions are reasonable politically, but they are not theoretically derived. The manuscript does not explain why these four dimensions should have equal importance, why they are sufficient dimensions of legitimacy, why alternative dimensions are excluded. Thus, VCTT is more a governance philosophy than a falsifiable theory.

 4. The literature should be a critical synthesis to identify a clear research gap. What exactly is unknown? For example, we already know elite quality is associated with governance, inequality affects trust, market concentration may affect political outcomes. The manuscript should demonstrate why existing institutional quality indices are insufficient and why VCTT solves a previously unresolved problem.

 5. Authors should have formal hypotheses. Without hypotheses, the logical structure is weak and post hoc.

 6. The study uses 22 countries in 5 years (2020–2024). This is a major limitation. Institutional legitimacy evolves slowly. Five years is insufficient to study institutional change, elite transformation, and legitimacy formation. The authors acknowledge this limitation but continue making broad institutional claims. Authors should expand the research period to 15-20 years.

 7. The authors admit elites influence institutions, institutions influence elites, market power influences institutions, institutions influence market power… However, no credible identification strategy exists. There are no instrumental variables, no dynamic panel methods, no causal design, no natural experiments. Consequently, coefficients cannot be interpreted causally.

 8. The construction of INST is arbitrary. The index is created by standardizing variables, assigning equal weights, averaging them. Why equal weights? There are no theoretical justification, no validation procedure, and no factor analysis to support the weighting structure. The index construction appears largely arbitrary.

 9. The manuscript relies heavily on hierarchical multiple imputation. However, the manuscript does not report percentage of missing values, missingness patterns, diagnostics, sensitivity to alternative imputation methods. Readers cannot assess how dependent results are on imputed observations.

 10. The manuscript frequently treats the Mundlak model as if it substantially addresses endogeneity. It does not. Mundlak controls for time-invariant heterogeneity, but it does not address reverse causality, simultaneity, omitted time-varying factors. Thus, the central identification problem remains unresolved

 11. Strange Gini results are not properly explained.  The manuscript finds negative within-country effects, positive between-country effects. This is potentially interesting. However, the explanation is speculative: moderate inequality may coexist with institutional stability. The authors should develop a theoretical framework explaining why inequality should improve legitimacy between countries.

**Conclusion:** The manuscript has potential as a conceptual or exploratory empirical contribution, but substantial revision is required before it can be accepted.

Is the work clearly and accurately presented and does it cite the current literature?

Partly

If applicable, is the statistical analysis and its interpretation appropriate?

Partly

Are all the source data underlying the results available to ensure full reproducibility?

Yes

Is the study design appropriate and is the work technically sound?

Partly

Are the conclusions drawn adequately supported by the results?

Yes

Are sufficient details of methods and analysis provided to allow replication by others?

Partly

Reviewer Expertise:

Institutional economics, Applied economics, Development economics

I confirm that I have read this submission and believe that I have an appropriate level of expertise to confirm that it is of an acceptable scientific standard, however I have significant reservations, as outlined above.

# Reviewer report: Reviewer response for version 2

**DOI:** 10.5256/f1000research.201165.r487792

**Published:** 2026-06-09

**Authors:** Syeda Azra Batool

**Affiliations:** 1 Bahahuddin Zakariya University, Multan, Pakistan

In Abstract the core regressors include the Elite Quality Index (EQx), market power (mark-up), the Gini coefficient, and GDP per capita. The writer is pretty much confused as interchangeably used the variables and their proxies which breaks the flow of the readers so it is suggested that

 1-         Should use inequality not Gini coefficient as variable label

 2-         Economic development not GDP per capita

 3-         Market power not mark up

 4-         Explain the Elite Quality Index (EQx)

 Tertiary education or human capital which one is your variable????? Again, confused which one you want to use? Please be clear.  With reference to introduction: Please write the flaws of already used measure of ‘ INST” : why it is grounded in VCTT? Why equal weights are given on what principle write with running text reference.   Literature review section is missing: regarding the independent and dependent variables relationship add this section with up-to-date studies. The contribution of the study is viable as new horizon is attempted to add by adding VCTT as proxy of “INST” variable. For policy implication a rigorous ground has been established, need more focused. Overall, the model is a real contribution as regards data source it is reliable and methodology is appropriate; Explain Mundlak model? Results are discussed not in effective manner.  need more reference based discussion.

**Conclusion:**  In light of the introduction data and methodology model construction results and discussion and the most importantly VCTT construct used for INST variable the manuscript is a good contribution. BUT the author is pretty much confused about use of variables interchangeably which is great demerit of any manuscript. if suggestions are well understood and incorporated, this can be a good addition in literature. Otherwise, the present form of manuscript is not able to be indexed in your prestigious journal. It needs great effort to rewrite it.  It has been decided to index the manuscript after many suggestions.

Is the work clearly and accurately presented and does it cite the current literature?

No

If applicable, is the statistical analysis and its interpretation appropriate?

No

Are all the source data underlying the results available to ensure full reproducibility?

Yes

Is the study design appropriate and is the work technically sound?

Yes

Are the conclusions drawn adequately supported by the results?

Yes

Are sufficient details of methods and analysis provided to allow replication by others?

No

Reviewer Expertise:

Economics and business

I confirm that I have read this submission and believe that I have an appropriate level of expertise to confirm that it is of an acceptable scientific standard, however I have significant reservations, as outlined above.

# Reviewer report: Reviewer response for version 2

**DOI:** 10.5256/f1000research.201165.r484853

**Published:** 2026-05-23

**Authors:** Kurniawan Arif Maspul

**Affiliations:** 1 Al Madinah International University, Kuala Lumpur, Malaysia

The revised manuscript demonstrates a meaningful and commendable evolution from the previous version. It is evident that the authors have engaged seriously and respectfully with the earlier review process, and the result is a substantially more coherent, transparent, and intellectually mature contribution. The clarification of the temporal scope, the explicit acknowledgment of the limitations associated with the short panel dimension, and the more cautious interpretation of causality significantly strengthen the credibility of the study.

 In particular, the revised framing around “robust associations” rather than deterministic causal claims reflects admirable academic discipline and methodological honesty. This gives the article greater integrity and makes its conclusions more persuasive rather than less.

 What makes this work particularly compelling is its ambition to bridge normative political theory with empirical institutional economics in a way that remains operational and measurable. The VCTT framework now feels far more grounded conceptually, especially after the expanded discussion on institutional persistence, state capacity, and elite structures. The article succeeds in capturing something profoundly human beneath the econometric architecture: the fragile relationship between power, fairness, and public belief in institutions.

 The discussion surrounding value-creating versus extractive elites is especially powerful because it transcends abstract theory and resonates with contemporary global anxieties regarding democratic erosion, concentration of wealth, and declining institutional trust across societies.

 The revised methodological explanations are also notably improved. The additional transparency regarding the construction of the INST index, the equal-weighting rationale, the PCA robustness check, and the hierarchical multiple imputation procedure substantially enhances reproducibility and reader confidence. The authors now communicate the econometric strategy with much greater precision and intellectual humility. The incorporation of the Mundlak decomposition as a mechanism to distinguish within-country and between-country dynamics is particularly insightful and gives the paper analytical depth that many cross-country institutional studies often lack.

 One of the most intellectually stimulating aspects of the manuscript is the nuanced interpretation of inequality. Rather than treating inequality as uniformly destructive in every context, the paper carefully differentiates between rising inequality within countries and historically embedded inequality across development trajectories. This subtle distinction reflects a sophisticated understanding of political economy and avoids simplistic moral or economistic binaries. The discussion here could become one of the paper’s most cited contributions because it opens a broader debate about whether legitimacy depends not only on distributive outcomes themselves, but also on perceptions of mobility, procedural fairness, and institutional responsiveness.

 The paper also deserves recognition for integrating market concentration into legitimacy analysis through the markup variable. This is an innovative and timely direction. In many contemporary democracies, citizens increasingly perceive that institutions are no longer neutral arbiters but systems disproportionately influenced by concentrated corporate and financial power. By empirically linking market concentration with institutional erosion, the article touches on a deeply important structural transformation occurring globally. This aspect gives the paper relevance beyond economics and political science, extending into governance studies, democratic theory, and even social psychology.

 At the same time, there remains room for one final layer of enrichment that could elevate the manuscript even further. While the paper discusses legitimacy primarily through structural and institutional dimensions, future iterations could briefly engage with the emotional and psychological foundations of legitimacy itself. Trust is not merely a rational evaluation of institutional efficiency; it is also relational, historical, and affective. Citizens often tolerate imperfect institutions when they feel represented, heard, and protected, while even economically successful systems can experience legitimacy crises when populations perceive humiliation, exclusion, or invisibility. Integrating a small reflection on the emotional sociology of trust could add remarkable interdisciplinary richness to the VCTT framework.

 Another promising avenue would be a deeper reflection on digital-era legitimacy. Transparency, voice, and trust are increasingly mediated through algorithmic systems, digital platforms, and information asymmetries shaped by technology corporations. The relationship between elite power and institutional legitimacy may now be evolving beyond traditional political and economic elites into data-driven informational elites. Even a brief acknowledgment of this transformation could position the article at the frontier of contemporary institutional research.

 Moreover, this revised article stands as a thoughtful, ambitious, and emotionally resonant contribution to the literature on institutions and political economy. It combines theoretical breadth with empirical rigour while maintaining a commendable degree of caution and self-awareness. The authors should be genuinely recognised for their willingness to refine the manuscript so substantially in response to critique. The study now possesses not only stronger methodological foundations but also a clearer intellectual identity and greater scholarly maturity. I have no major further concerns, and I believe this work has the potential to stimulate important discussions on legitimacy, inequality, governance, and the future architecture of democratic institutions.

Is the work clearly and accurately presented and does it cite the current literature?

Partly

If applicable, is the statistical analysis and its interpretation appropriate?

Yes

Are all the source data underlying the results available to ensure full reproducibility?

Partly

Is the study design appropriate and is the work technically sound?

Partly

Are the conclusions drawn adequately supported by the results?

Yes

Are sufficient details of methods and analysis provided to allow replication by others?

Partly

Reviewer Expertise:

Political economy; institutional economics; comparative politics; inequality and development; governance and public policy; applied econometrics (panel data analysis).

I confirm that I have read this submission and believe that I have an appropriate level of expertise to confirm that it is of an acceptable scientific standard.

# Reviewer report: Reviewer response for version 1

**DOI:** 10.5256/f1000research.192849.r462983

**Published:** 2026-02-28

**Authors:** Kurniawan Arif Maspul

**Affiliations:** 1 Al Madinah International University, Kuala Lumpur, Malaysia

This paper tackles a critically relevant and timely question in political economy: how can institutional legitimacy be conceptualised, quantified, and empirically related to elite structure, market power, and inequality? The study contributes significantly to ongoing debates regarding inclusive versus extractive institutions by transforming the Voice-Choice-Transparency-Trust (VCTT) paradigm into a composite institutional index and testing it in a panel econometric environment. Its attempt to integrate macrohistorical institutional theory with replicable quantitative tools increases its scientific significance.

 The incorporation of elite quality measurements and market mark-ups into institutional legitimacy analysis, in particular, provides a unique perspective that can drive additional multidisciplinary study in economics, political science, and governance studies.

 The title of the article is broadly suitable and accurately reflects the core constructs under investigation. It clearly signals the central theme—building institutional legitimacy—and identifies the main explanatory dimensions: elites, markets, and inequality, framed through the VCTT model.

 The abstract is generally thorough and well-structured, including background, methodology, results, and conclusions in a consistent order. It effectively conveys the composite index's development, empirical technique, and key conclusions. However, it would benefit from a more precise definition of the panel's temporal scope and relatively limited time horizon (2000-2004), which is critical for assessing the strength of causal assertions. Furthermore, the abstract might briefly include the use of multiple imputation and within-between decomposition inside the Mundlak framework, as these are methodological advancements discussed in the manuscript. A little strengthening of the inequality discussion—clearly distinguishing between within-country and between-country effects—would also avoid potential uncertainty for readers perusing the summary.

 Overall, the manuscript is scientifically sound and methodologically rigorous. The econometric technique is adequate for a small, imbalanced panel, and the use of Driscoll-Kraay standard errors, Mundlak adjustments, and robustness diagnostics reveals an understanding of frequent panel-data issues. The theoretical framework is solidly grounded in known literature, and the empirical findings are interpreted with caution rather than exaggeration. Nonetheless, certain internal consistency issues need to be addressed. For example, there appears to be a mismatch between the reported sample period (2000-2004 in some sections and 2020-2024 in the Methods section), which must be adjusted to avoid confusion.

 Clarifying the rationale for the short time range and identifying limitations associated with tiny T would improve the study's scientific credibility. Furthermore, a more in-depth examination of potential endogeneity between institutional quality and elite structure—aside from the GMM mentioned in the introduction—would strengthen the causal interpretation.

 The references are extensive and generally relevant, ranging from foundational works (North, Acemoglu & Robinson, Weber, Lipset) to more contemporary contributions on inequality, market concentration, and institutional trust. The inclusion of De Loecker et al. (2020), new research on inequality and trust (e.g., Palmisano & Sacchi, 2024), and the Elite Quality Index reports demonstrate a commitment to current scholarship. However, the text could be improved by including more current panel-causation or dynamic institutional research published beyond 2020, particularly those that investigate reverse causality between institutions and economic structure.

 Additional empirical contributions from journals such as World Politics, Comparative Political Studies, and the Journal of Development Economics that explicitly model institutional persistence would provide more context. A brief review of the literature on state capacity measurement (e.g., Hanson & Sigman or related datasets) could help improve the conceptualisation of legitimacy.

 The English language quality is generally adequate for scholarly communication. The manuscript shows an excellent knowledge of academic terminology and theoretical explication. Some paragraphs, particularly in the theoretical sections, are dense and might use a little stylistic tightening to improve readability and flow.

 In general, this is a serious and ambitious contribution that aims to go beyond descriptive institutional typologies to quantitative, policy-relevant indices of legitimacy. Its key merits are the conceptual integration of elite behaviour and market structure into legitimacy analysis, as well as the methodological transparency. Strengthening internal consistency, clarifying temporal scope, and addressing endogeneity problems will all considerably improve its robustness and impact. With these improvements, the paper has the potential to contribute significantly to discussions about institutional development, governance quality, and the political economy of inequality.

*Is the work clearly and accurately presented and does it cite the current literature?*

**Partly.**

 The work is typically well-structured and logically cohesive, and it draws significantly on fundamental and recent literature in political economy, institutional theory, inequality, and market power. The theoretical framework is clearly described, and the majority of statements are supported by relevant reference. However, there are some internal inconsistencies, particularly in the time range of analysis (2000-2004 versus 2020-2024 in the Methods section), which reduce clarity and precision. Furthermore, while the literature coverage is comprehensive and robust, a more in-depth engagement with recent empirical panel research on institutional persistence and causal identification would improve alignment with current scholarship.

*Is the study design appropriate and is the work technically sound?*

**Partly.**

 The overall study design is adequate for the research objective, and the use of fixed effects, Mundlak correlated random effects, and Driscoll-Kraay robust standard errors demonstrates strong econometric reasoning for short panel data. The use of multiple imputation and within-between decomposition is methodologically sound. However, the short time dimension (T=5) limits dynamic inference, and while endogeneity is recognised, the issue of identification technique and causal direction may be expanded further. Addressing these concerns directly would improve technical robustness.

*Are sufficient details of methods and analysis provided to allow replication by others?*

**Partly.**

 The manuscript explicitly identifies data sources and presents a general econometric specification, which aids conceptual replication. However, full replication would necessitate more precise information about variable construction (for example, the actual weighting technique for the INST index, standardisation procedures, imputation model specification, ARIMA parameterisation, and software code). While the data is publicly accessible, the lack of a consolidated dataset or replication code hinders comprehensive procedural reproducibility.

*If applicable, is the statistical analysis and its interpretation appropriate?*

**Yes.**

 The statistical procedures used are acceptable for the data format, and coefficient interpretation is generally conservative and consistent with stated estimates. In the Mundlak model, the distinction between within-country and between-country effects is especially well understood. The authors avoid making excessive causal assertions, and the debate demonstrates an understanding of structural subtlety, particularly in terms of inequality's uneven impacts.

*Are all the source data underlying the results available to ensure full reproducibility?*

**Partly.**

 All underlying datasets are freely available from respected repositories (e.g., V-Dem, WGI, SWIID, Elite Quality Index, World Bank, OECD), promoting transparency. However, because no harmonised datasets or replication files have been deposited, and given the use of imputation and composite index building, exact replication of the analytical dataset would necessitate more documentation. Providing replication materials would dramatically improve reproducibility.

*Are the conclusions drawn adequately supported by the results?*

**Yes.**

 The results are broadly compatible with the empirical data. The authors correctly stress the positive relationship between value-creating elites and institutional legitimacy, the negative relationship with prolonged market concentration, and the nuanced impact of inequality. The claims are appropriate to the statistical evidence presented, and the policy implications are properly inferred from the findings without exceeding the empirical scope.

Is the work clearly and accurately presented and does it cite the current literature?

Partly

If applicable, is the statistical analysis and its interpretation appropriate?

Yes

Are all the source data underlying the results available to ensure full reproducibility?

Partly

Is the study design appropriate and is the work technically sound?

Partly

Are the conclusions drawn adequately supported by the results?

Yes

Are sufficient details of methods and analysis provided to allow replication by others?

Partly

Reviewer Expertise:

Political economy; institutional economics; comparative politics; inequality and development; governance and public policy; applied econometrics (panel data analysis).

I confirm that I have read this submission and believe that I have an appropriate level of expertise to confirm that it is of an acceptable scientific standard, however I have significant reservations, as outlined above.

# Response

**Published:** 2026-05-04

**Authors:** William Pacheco

**Affiliations:** Economics, University Anahuac Mexico, Huixquilucan de Degollado, State of Mexico, Mexico

Dear Reviewer,

 We sincerely thank you for your careful and constructive evaluation of our manuscript. We are grateful for your recognition of the relevance and potential contribution of the paper, particularly its attempt to operationalise institutional legitimacy through the VCTT framework and to connect this with elite structure, market power, and inequality.

 We also appreciate the specific reservations you have raised regarding the temporal scope of the analysis, internal consistency, endogeneity, methodological detail, reproducibility, and engagement with recent empirical literature. These comments are very helpful and will guide the next revision of the manuscript.

 In response, we will revise the paper to address the issues identified. In particular, we will clarify and correct the temporal scope of the analysis, strengthen the abstract, expand the discussion of endogeneity and causal limitations, provide more detail on index construction, standardisation, imputation, and model specification, and improve the explanation of the short panel structure. We will also refine the discussion of within-country and between-country effects and expand the literature review where relevant, especially in relation to institutional persistence, state capacity, and panel-data approaches in comparative political economy.

 We thank the reviewer again for the thoughtful feedback. We believe these suggested changes will help improve the clarity, transparency, and robustness of the manuscript.
